# Impact of aging on acute myeloid leukemia epidemiology and survival outcomes: A real-world, population-based longitudinal cohort study

**DOI:** 10.1371/journal.pone.0300637

**Published:** 2024-05-21

**Authors:** Hyun Jin Han, Kyungson Choi, Hae Sun Suh

**Affiliations:** 1 Department of Regulatory Science, Graduate School, Kyung Hee University, Seoul, Republic of Korea; 2 Institute of Regulatory Innovation through Science (IRIS), Kyung Hee University, Seoul, Republic of Korea; 3 College of Pharmacy, Kyung Hee University, Seoul, Republic of Korea; Istanbul University-Cerrahpaşa, Cerrahpaşa Faculty of Medicine, TURKEY

## Abstract

Acute myeloid leukemia (AML) is a severe and fatal form of leukemia that is prevalent in the older population. In this longitudinal retrospective study, we investigated the epidemiology and survival rates of patients diagnosed with de novo acute myeloid leukemia in South Korea from Jan 1, 2011, to Aug 31, 2020. We used real-world data from the Health Insurance Review and Assessment Service database. We observed an increase in the number of acute myeloid leukemia cases, with age-specific incidence rates escalating in older patients. In contrast a long-term decrease from 1.94 to 1.77 per 100,000 individuals was found in the age-standardized incidence rates. Meanwhile, age-standardized prevalence rates ascended from 8.93 to 9.67 per 100,000 individuals, with a remarkable increase in the age-specific prevalence rate for those aged 80 years and above. Survival rates were notably better in younger or treated patients, and in those who underwent Hematopoietic stem cell transplantation. The time of diagnosis did not affect the survival of patients younger than 65 years. However, the most recent survival rates were significantly lower for patients 65 or older, as shown in the unadjusted Cox survival analysis. After adjustments in the analysis, it was found that the overall survival rates of the most recently diagnosed group improved significantly compared with those diagnosed earlier, with a hazard ratio of 0.90 (95% confidence interval, 0.84–0.97). This improvement may potentially be influenced by the enhanced treatment alternatives available for newly diagnosed older patients aged 65 years or older. In conclusion, aging appears to fuel an increase in the number of acute myeloid leukemia cases and mortality. Further studies are warranted to understand the impact of aging on acute myeloid leukemia treatment outcomes and devise efficacious care strategies for older patients.

## Introduction

Acute myeloid leukemia (AML) is a heterogeneous hematologic malignancy characterized by aggressive clonal proliferation of myeloid blasts in the bone marrow and blood, with a poor prognosis [1]. AML is the predominant form of acute leukemia diagnosed in adults, and its incidence and mortality are even higher in older populations [2,3]. According to the Surveillance, Epidemiology, and End Results (SEER) program, leukemia is projected to account for 3.0% of all new cancer diagnoses, and 3.9% of all cancer-related deaths in 2023 [4].

As the global population ages, the incidence and burden of AML are gradually increasing, and this trend is becoming more pronounced in developed countries. This is also evident in Asia, particularly in the high-income Asia Pacific region where, the incidence of leukemia is rising rapidly, with an estimated annual percentage increase of 0.74 from 1990 to 2017 [5]. However, there is a notable dearth of research on the epidemiology of AML in Asia, which does not adequately reflect the prevailing real-world situation [6]. To the best of our knowledge, there have been no nationwide, population-based, real-world epidemiological, or survival studies on de novo AML, even though patients with de novo AML exhibit distinct etiologies and survival rates compared with those with other types of AML.

AML is characterized by significant variations in treatment approaches, disease management strategies, and prognoses based on age. Different populations exhibit heterogeneity in treatment methods, rates, and outcomes. Long-term follow-up registry data of leukemia patients in Osaka, Japan, showed an improvement in the 5-year survival rate of AML in children and adolescents from 7.0% to 77.0% and from 5.2% to 66.5% respectively from 1975–2011 [7]. However, older AML patients who are 65 years or older showed a poor 5-year survival rate of 12.5% during 2009–2013 with a treatment rate of approximately 50%, based on the SEER database [8,9]. A nationwide population study in the Netherlands reported a similar survival rate of 14% between 2007–2012 [10]. According to more recent data from the SEER-Medicare database from 2008–2015, the treatment rate, encompassing both AML treatment and supportive care, rose to 69.04%, while the rate of AML treatment alone stood at 27.17% [11]. These results indicate an unmet need for AML treatment in older individuals. Since the late 2000s, hypomethylating agent (HMA) therapies such as azacytidine and decitabine for older patients with newly diagnosed AML, and those who are unable to undergo intensive chemotherapy have been implemented. This has raised expectations of enhanced treatment rates and outcomes for those aged 65 years or older, as demonstrated in clinical trials [12–15]. However, the treatment effects of monotherapy are modest at best and are unlikely to have significantly altered survival outcomes [16,17]. Therefore, combination regimens such as venetoclax plus HMA were introduced in 2019. The standard of care for newly diagnosed AML still remains with HAM therapies in South Korea until early 2023, and there are concerns about the limited improvement of AML survival in the elderly.

This study aimed to examine the epidemiology and characteristics of patients with de novo AML over the past decade, using population-based, real-world data from South Korea. Additionally, we aimed to compare the observed survival rates across different subgroups and diagnosis periods, with a particular focus on older age groups.

## Materials and methods

### Study design

This longitudinal retrospective cohort study was based on the Korean population utilizing the databases of the patients with diagnosed AML from January 1, 2008, to August 31, 2020. The detailed study design and settings are shown in S1 Fig. We adhered to the guidelines provided by the Strengthening the Reporting of Observational Studies in Epidemiology statement checklist [18].

### Study setting and participants

Eligible patients with AML were defined as those with a diagnosis corresponding to the International Classification of Diseases tenth revision (ICD-10) codes C92.00, C92.01, C92.08, C92.5, C92.6, C92.8, C93.0, C94.0, C94.2, and C94.4. As described in a previous study, de novo AML was defined as AML without a history of myelodysplastic syndrome (MDS), myeloproliferative disorder, or leukemogenic therapies [19]. The exclusion criteria for the de novo AML cohort were a prior history of secondary AML conditions, such as MDS, chronic myelomonocytic leukemia, and myeloproliferative neoplasia before the index date of first AML diagnosis. Due to the challenges in identifying previous disease histories and ensuring data stability, the first three years of the data were excluded from the study and defined as the pre-index period. Patients newly diagnosed with AML from January 1, 2011, to December 31, 2019, were selected for the incidence and prevalence analysis. For the survival outcomes analysis, de novo AML cases from January 1, 2011, to August 31, 2020, were included based on criteria that defined the study population.

### Data sources and measurement

The study population was extracted from the Health Insurance Review and Assessment Service (HIRA) database, which encompasses all Korean patients with AML, with a history of health insurance claims between January 1, 2008, and August 31, 2020 (data accessed May 10, 2022). The HIRA is a representative Korean population database established for the review of health insurance claims from the National Health Insurance Service (NHIS) database. South Korea operates a single-payer universal healthcare system wherein the NHIS comprehensively records all medical utilization. The integrity and completeness of this database enable its extensive use in a wide range of epidemiological and health policy studies [20,21]. Details of the database profile have been described elsewhere [22,23]. Given the comprehensiveness of the HIRA database, our data fully represents the South Korean population, and most patients with a history of a clinical diagnosis of AML were included in the study. Another advantage of this dataset is that it allows for the construction of longitudinal cohort studies owing to its continuous multi-year data. In our study, these database characteristics enabled us to reasonably categorize in-hospital deaths as mortality. Considering the severity of AML and the South Korean context, we anticipate that our classification of deaths will be comparatively accurate. However, worth noting that this could introduce a potential source of bias.

We sourced annual mid-year population statistics from the Korean Statistical Information Service (https://kostat.go.kr/ansk/) to calculate the incidence and prevalence rates.

The incidence and survival outcomes were estimated from the de novo AML cohort, while prevalence was derived from all prevalent population diagnosed with AML during the study period. Prevalent cases were defined as annual incident cases, and all patients diagnosed with AML in prior years. The annual incidence and prevalence of crude, age-specific, and age-standardized rates were measured. The survival time was measured in months from the first AML diagnosis to death.

### Ethical statement

The HIRA database was de-identified and encrypted to ensure confidentiality. Moreover, reassigned unique identification numbers were used to connect the database subsets from the NHIS. The study was conducted in accordance with the Declaration of Helsinki and approved by the Institutional Review Board (or Ethics Committee) of Pusan University (PNU IRB/2019_30_HR, March 16, 2019) for the waiver of informed consent.

### Variables

The study outcomes encompass annual incidence rates and observed overall survival (OS), analyzed across subpopulations and three diagnostic periods: January 1, 2011, to December 31, 2013 (period 1), January 1, 2014, to December 31, 2016 (period 2), and January 1, 2017, to August 31, 2020 (period 3). The observed OS hazard ratios (HR) were estimated and compared across periods, age groups, and treatment modalities, representing the prognostic factors available in the claims data.

We also examined several variables, including sex, age at diagnosis, history of hematopoietic stem cell transplantation (HSCT), treatment status, Charlson Comorbidity Index (CCI) scores, type of initial healthcare facility, and insurance type. The baseline characteristics were measured within a year of the index date. Age was stratified into ten-year increments from 20 to 79 years, with additional categories for those younger than 20 years and older than 80 years. The treatment guidelines were adapted from the American Cancer Society recommendations for the rapid initiation of AML therapy. Treated patients were defined as those receiving HSTC or systemic chemotherapy treatment within a 90-day window after the index date, or seven days before it, consistent with the drug regimens for AML available in Korea as per Ha et al. (2022) and the HIRA reimbursement guidelines [24].

### Statistical methods

We presented the baseline characteristics of the study population, including numerical and percentage data, across the three diagnostic periods. Continuous variables were analyzed using t-tests, while categorical variables were subjected to chi-square (χ2) tests.

The crude estimate of the annual incidence and prevalent cases was defined by the age group of 0–19 years, 10-year increment age groups from 20 to 70 years, and 80 years or older. The annual incidence per 100,000 people was age-adjusted to the 2011 Korean standard population for the age-standardized incidence rates (ASIRs). We calculated the ASIRs as follows: firstly, we estimated age-specific incidence rates for each age group by dividing the number of incident deaths by the population of that age group and then multiplying the result by 100,000. Subsequently, each age-specific rate underwent an additional adjustment. We multiplied these rates by the proportion of the 2011 Korean population corresponding to the respective age group, commonly referred to as the standard population weight. Direct standardization was performed using the 2011 Korean mid-year population as a reference.

To assess the observed survival outcomes, the cohort was followed up until death or the final data cutoff date of August 31, 2020, whichever came first. Kaplan-Meier analysis was used to estimate survival rates, and univariate Cox regression was used to investigate the associations between survival and prognostic factors of interest. For the comparison of survival rates across different AML diagnosis periods, we conducted an adjusted Cox survival analysis, incorporating variables including sex, age, CCI, HSCT, and insurance type. The log-rank test revealed significant differences in survival among subgroups.

## Results

### Participants and baseline characteristics

Among 56,224 patients diagnosed with myeloid leukemia between January 2008 and August 2020, 18,995 met the inclusion criteria for AML diagnosis (Table 1). The final study cohort was established after excluding patients with any history of secondary AML prior to their first AML diagnosis and those diagnosed during the pre-index period. The cohort selection process for estimating the observed survivals is illustrated in S2 Fig. In Korea, from 2011 to 2020, there were 9,495 patients with de novo AML. For the incidence estimation, patients with de novo AML from 2011 to 2019 were 8,906. In Korea, from 2011 to 2020, a total of 9,495 patients were identified with de novo AML. For the purposes of estimating incidence, the number of patients with de novo AML from 2011 to 2019 was 8,906.

**Table 1:** Characteristics of patient with de novo AML in South Korea from 2011 to 2020.

	Total N = 9,495	Diagnosis in 2011–2013 (Period 1) N = 2,840	Diagnosis in 2014–2016 (Period 2) N = 2,937	Diagnosis in 2017–2020 (Period 3) N = 3,718	*p*
**Sex, n (%)**					
Male	5,132 (54.05)	1,526 (53.73)	1,595 (54.31)	2,011 (54.09)	0.907
Female	4,363(45.95)	1,314 (46.27)	1,342 (45.69)	1,707 (45.91)	
**Age at diagnosis, n (%)**					
<20	833 (8.77)	316 (11.13)	275 (9.36)	242 (6.51)	<.0001
20–29	569 (5.99)	177 (6.23)	173 (5.89)	219 (5.89)	
30–39	849 (8.94)	292 (10.28)	261 (8.89)	296 (7.96)	
40–49	1,242 (13.08)	394 (13.87)	371 (12.63)	477 (12.83)	
50–59	1,736 (18.28)	499 (17.57)	569 (19.37)	668 (17.97)	
60–69	1,806 (19.02)	529 (18.63)	521 (17.74)	756 (20.33)	
70–79	1,774 (18.68)	492 (17.32)	561 (19.1)	721 (19.39)	
≥80	686 (7.22)	141 (4.96)	206 (7.01)	339 (9.12)	
**HSCT**^a^, **n (%)**					
Yes	2,905 (30.6)	875 (30.81)	960 (32.69)	1,070 (28.78)	0.003
No	6,590 (69.4)	1,965 (69.19)	1,977 (67.31)	2,648 (71.22)	
**Treated**^b^, **n (%)**					
Yes	6,919 (72.87)	2,015 (70.95)	2,212 (75.31)	2,692 (72.4)	0.001
No	2,576 (27.13)	825 (29.05)	725 (24.69)	1,026 (27.6)	
**CCI**^c^, **n (%)**					
0	965 (10.16)	314 (32.54)	289 (29.95)	362 (37.51)	<.0001
1	1,486 (15.65)	528 (35.53)	479 (32.23)	479 (32.23)	
2	1,715 (18.06)	520 (30.32)	535 (31.2)	660 (38.48)	
≥3	5,329 (56.12)	1,478 (27.74)	1,634 (30.66)	2,217 (41.6)	
**Type of HCO** ^d^					
Tertiary	6,909 (72.76)	2,096 (73.8)	2,180 (74.23)	2,633 (70.82)	0.107
Secondary	11,975 (20.8)	584 (20.56)	573 (19.51)	818 (22.00)	
Others	611 (6.43)	160 (5.63)	184 (6.26)	267 (7.18)	
**Insurance Type**					
NHI^e^	9,041 (95.2)	2,693 (94.8)	2,806 (95.5)	3,542 (95.3)	0.762
Others^f^	454 (4.8)	147 (5.2)	131 (4.5)	176 (4.7)	

Note that the cumulative percentage may not equate to 100% as a result of rounding.

^a^ HSCT: Hematopoietic Stem Cell Transplantation.

^b^ Treated with systemic chemotherapy or HSCT.

^c^ CCI: Charlson comorbidity index.

^d^ HCO: Healthcare organization.

^e^ National Health Insurance.

^f^ Included 433 Medicare and 21 veterans care beneficiaries.

We noted a steady increase in de novo AML cases, accompanied by a markedly higher rate in older patients than in younger patients. The proportion of patients aged 60 years or older varied, starting from 40.92% in period 1, increasing to 42.85% in period 2, and reaching 48.84% in period 3. The sex distribution among AML patients remained consistent throughout the study period. A statistically significant rise was observed in the proportion of patients with a CCI score of 3 or higher, escalating from 27.74% at the beginning of the study period to 41.6% by its conclusion. Furthermore, the mean CCI score at each diagnostic period significantly differed (p<0.0001). The rate of patients with de novo AML undergoing chemotherapy and HSCT showed notable fluctuations across the periods, peaking in period 2 before experiencing a minor decline in period 3. The number of patients who received no treatment was 27.13% during the study period, and there was a significant decrease in the treatment rate in periods 2 and 3 compared with that in period1. Over 70% of patients with de novo AML were diagnosed at tertiary healthcare facilities, and approximately 95% were consistently insured under the National Health Insurance scheme across all periods.

### Incidence of de novo AML

**Fig 1:**
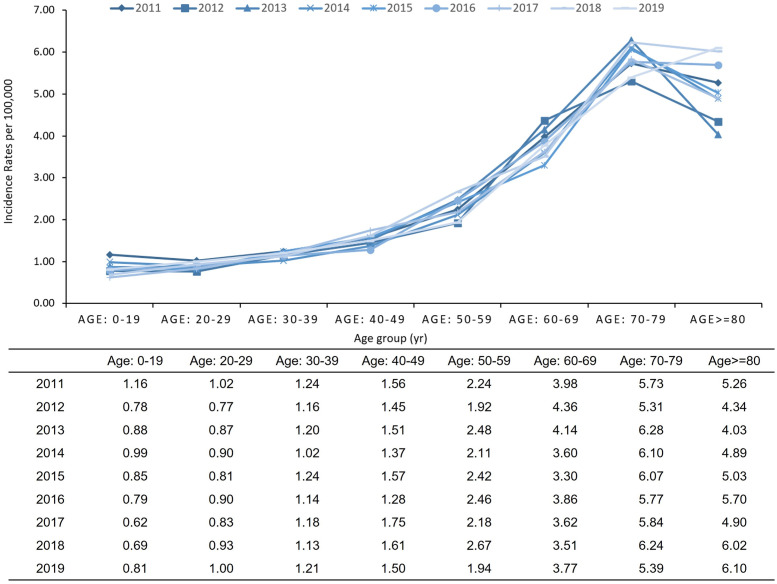
Age-specific incidence rate of de novo AML from 2011 to 2019 in Korea.

The ASIR of de novo AML per 100,000 individuals showed minor fluctuations, but a long-term minimally declining trend from 1.94 to 1.77 per 100,000 individuals from 2011 to 2019. In contrast, the crude incidence rate increase from 1.94 to 2.03 per 100,000 people over the same period. Notably, there was a marked increase in the incidence rates among patients aged 60 years or older (Fig 1). During the longitudinal follow-up from 2011 to 2019, patients aged 80 years or older experienced a 15.95% overall and monotonously increasing trend of incidence rates. However, individuals in their 60s demonstrated a declining trend in incidence rates during the study period from 3.98 to 3.77 per 100,000 from 2011 to 2019. Notably, there was an observable reduction in the overall incidence rates among patients younger than 60 years and a sharp decrease among those younger than 20 years (-30.32%).

### Prevalence of de novo AML

**Fig 2:**
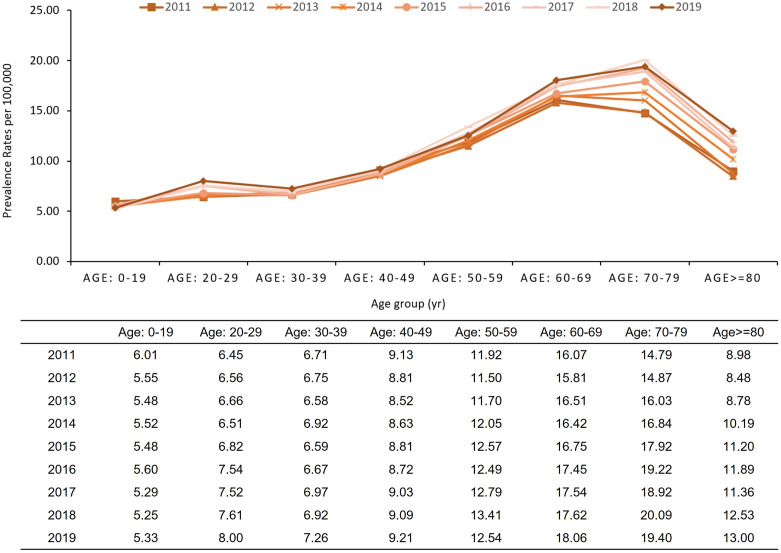
Age-specific prevalence cases of de novo AML from 2011 to 2019 in Korea.

From 2011 to 2019, the age-standardized prevalence rates (ASPRs) of de novo AML increased from 8.93 to 9.67, and the crude prevalence rates increased from 8.93 to 10.50 per 100,000 individuals (Table 2). Age-specific prevalence rates were significantly higher in older compared to younger patients with AML. Notably, the highest increase rates were observed in patients aged 80 years or older at 44.75%, followed by those in their 70s at 31.19%, and in their 20s at 24.08% (Fig 2).

**Table 2:** Prevalence and incidence rates per 100,000 individuals with de novo AML.

	Prevalence		Incidence	
	Crude	Age-standardized	Crude	Age-standardized
2011	8.93	8.93	1.94	1.94
2012	8.78	8.71	1.75	1.73
2013	8.94	8.78	1.95	1.89
2014	9.21	8.96	1.86	1.77
2015	9.51	9.14	1.94	1.82
2016	9.87	9.39	1.96	1.80
2017	10.03	9.45	1.95	1.77
2018	10.37	9.63	2.11	1.87
2019	10.50	9.67	2.03	1.77

### Comparisons of survival outcomes

**Fig 3:**
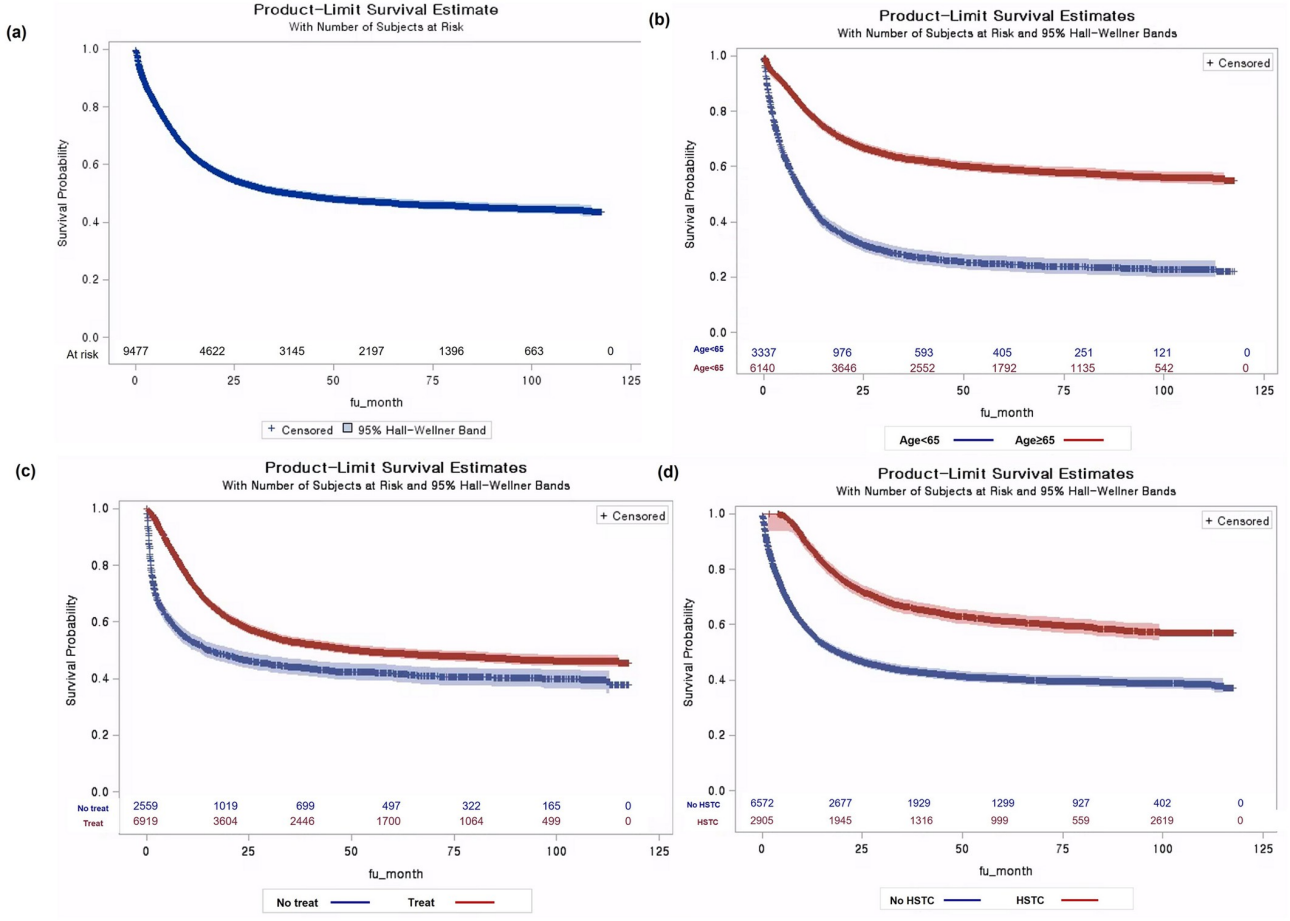
Overall survival (OS) of patient with newly diagnosed AML in South Korea, from 2011 to2020 by subpopulation: (a) Overall population, (b) OS by Age group, (c) OS by Treatment, (d) OS by HSTC.

The mean follow-up period of patients with de novo AML was 33.62 months (standard deviation:34.13 months) and the median observed OS was 38.6 months (95% confidence interval (CI) 33.77–44.5 months). We compared OS by age group, treatment status, and HSCT experience (Fig 3). The younger population (age <65 years) survived significantly longer than did the older population (log-rank test p<0.0001). Although the younger patient cohort did not reach the median observed OS by the end of the study follow-up period, the older cohort achieved their median OS within 12 months of diagnosis. The OS HR for patients aged 65 years or older compared to the younger population was 2.99 (95% CI, 2.92–3.06). Treatment status and HSCT significantly influenced the observed OS rates, as indicated by a log-rank test p-value of <0.0001. For the untreated group and HSCT recipients compared to the reference group, the OS HRs were 2.04 (95% CI, 1.97–2.10) and 0.45 (95% CI, 0.38–0.51), respectively.

**Fig 4:**
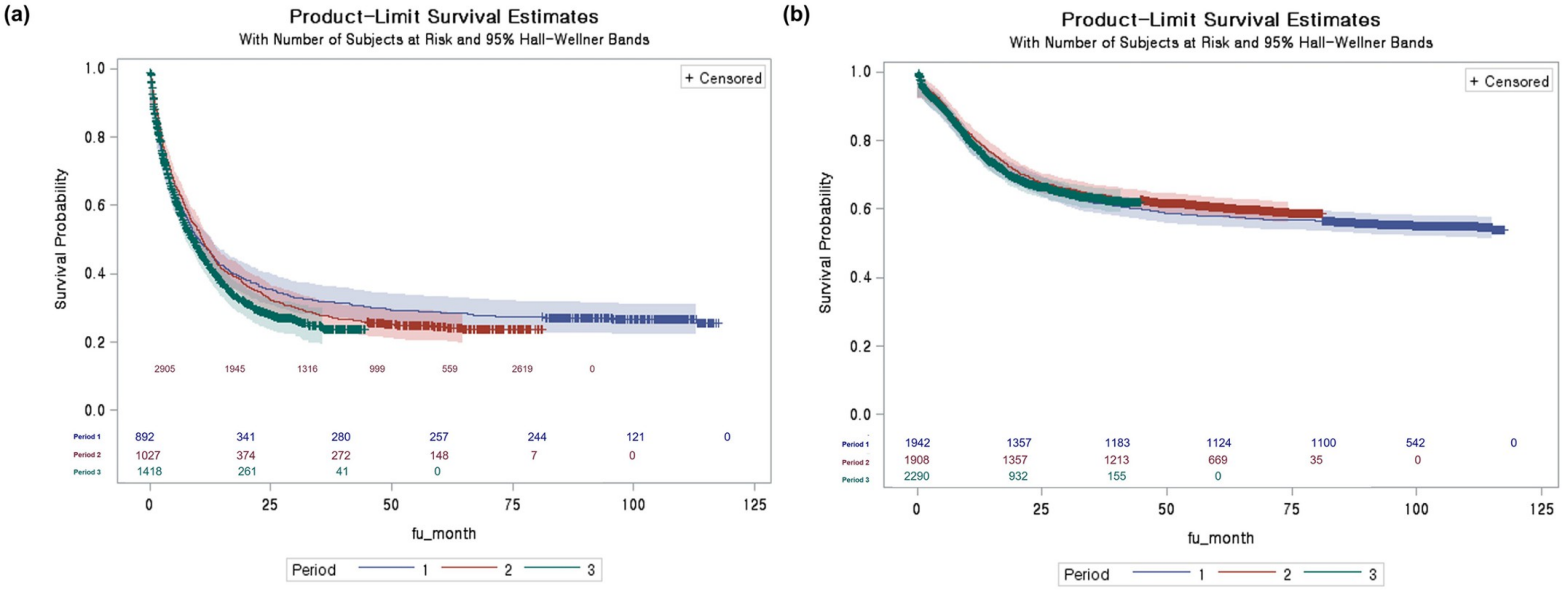
Overall survival of patient with de novo AML in South Korea, from 2011 to 2020 by diagnosis period: (a) age≥ 65 and (b) age <65.

The change in the observed OS according to the time of diagnosis is presented in Fig 4. Older patients aged 65 years or older diagnosed in periods 2 and 3 showed a significantly worse deterioration in OS compared with those diagnosed in period 1 (p = 0.0003). However, younger patients without changes in the reimbursed treatment during the study period showed no changes in the OS according to the diagnosis period (p = 0.2532). The adjusted Cox survival analysis showed an improvement in observed OS during period 3, with an HR of 0.90 (95% CI, 0.84–0.97). This result indicates a statistically significant reduction in the risk of mortality among the patients in the third period compared to the reference period.

## Discussion

To the best of our knowledge, this is the first population-based study to report the incidence, prevalence, and treatment outcomes in patients diagnosed with de novo AML across population subgroups. Several recent epidemiological studies have been conducted at a global level and in countries within North America, Europe, and Australia. However, there has been limited population-based research in high-income Asian countries [3,5,25–31], particularly concerning the de novo type of AML. A recent population-based AML epidemiological and survival study in Korea reported on the incidence of AML between 1999 and 2012 [32]. A study in Taiwan scrutinized the epidemiology, treatment patterns, healthcare utilization, and cost of AML treatment from 2006 to 2015; however, this study did not provide details on de novo AML [6].

Our study demonstrated a decreasing trend in the ASIR of de novo AML from 2011 to 2019, in contrast to an increasing trend in crude incidence rates during the same period. The annual ASIRs of de novo AML in Korea between 2011 and 2019 ranged from 1.94 to 1.77 per 100,000. This rate is considerably lower than that reported in the US, Canada, Australia, Sweden, and Denmark, representing AML, but not de novo AML [3,5,25–31]. A study of de novo AML in England reported an annual incidence of 6.5 per 100,000, including APL, in a cohort from 1988 to 1991 [33]. Therefore, cautious comparisons are necessary as the definitions of AML vary across studies, with some including APL [3] or adopting even broader definitions, comprising AML with recurrent genetic abnormalities, myeloid sarcoma, myeloid proliferations related to Down Syndrome, and therapy-related myeloid neoplasms [6]. These results confirm the lower incidence and decreasing ASIR trend of AML observed in high-income Asian countries. The trend of age-specific incidence rates across age groups is quite similar to that in the US and Sweden, with an exponential increase with a peak at 70–79 years and a decrease after 80 years or older [34]. Another notable finding from our study was the increasing trend in the annual crude incidence rates (from 8.93 to 9.67 per 100,000) and ASPRs (from 8.93 to 10.50 per 100,000 individuals) for de novo AML from 2011 to 2019, despite the decrease in ASIRs.

Another key finding was that the ASPRs of patients with de novo AML in Korea were lower than those in Western countries. This trend aligns with the incidence results, although a direct comparison is challenging owing to the differences in AML types. According to the SEER data, the estimated prevalence in the USA was 19 per 100,000 individuals. Moreover the estimated overall prevalence in Sweden was 13.7 per 100,000, while that in other Scandinavian countries ranged from 12.2 to 16.8 per 100,000 individuals [3,30]. The ASPR was higher in populations aged 60 years or older than younger population, with a particularly significant increase in age-specific prevalence of 44.75% among those aged 80 years and older between 2011 and 2019. Prevalence studies of AML are even more limited because registry-based or fragmented claims-based analyses make accurate disease prevalence estimations more challenging [3]. Our population-based claims data estimations, derived from a universal national healthcare system, overcome these limitations.

To the best of our knowledge, there is limited evidence offering population-based, real-world insights into survival time for de novo AML, especially for subgroups spanning different age groups, treatment modalities, and periods of diagnosis. Our data revealed a median OS estimate for de novo AML of 38.6 months, suggesting a notably long survival time at the population level. A study focused on patients aged 60 or older with de novo AML, treated with 7 + 3 (cytarabine + anthracycline), showed an OS of 14 months (95% CI:11.0, not estimated) [35]. However, more real-world studies on the survival rates in a broader population are needed.

Our findings confirm and uniquely present the HRs of important factors of de novo AML mortality. These results align with prior studies highlighting increased HRs for populations aged 60 or older. However, only some of these past studies have provided the OS HR of the older population compared with that of the younger population [8,36,37]. AML is well known for its poor prognosis and limited improvement in treatment outcomes in older patients [2]. The rates of our patients with treated de novo AML are similar to those of a previous study, with a 71% treatment rate [38]. However, aging and intensive treatment rates are interrelated factors and require careful attention with the complex source of unmet treatment needs in this aging era. Intensive therapy, including chemotherapy and HSCT, can be influenced by factors related to patients, disease-specific factors, and socioeconomic characteristics. Importantly, patients with de novo AML are more likely to receive intensive treatment than secondary AML patients, and the treatment rate for t-AML is only approximately 66%, even though most t-AML patients are younger than 65 years [3]. The untreated cohort of newly diagnosed patients with AML, which tends to increase with age, comorbidities, and socio-economic challenges, warrants greater clinical attention [39,40]. Therefore, more attention should be paid to care and treatment strategies, especially in older patients, to improve survival.

The period of diagnosis did not significantly influence OS across age groups. However, our adjusted Cox analysis showed an approximately 10% improvement in OS among the most recently diagnosed patients with de novo AML compared to that from earlier periods. This improvement may be associated with Korea’s recent expansion of reimbursed treatments for older patients with de novo AML, including the introduction of two hypomethylating agents: decitabine in December 2013 and azacytidine in September 2017. These survival improvements are also attributed to advanced supportive care such as hematopoietic cell stimulating agents, antimicrobials, and improved transfusion care [41,42]. The HIRA database, which includes all Korean patients with AML with a history of health insurance claims, is a powerful population-based dataset; however, it has several limitations. First, the lack of molecular or cytogenetic information cannot fully address prognostic factors that may affect survival. Second, the nature of claims data could induce under- or over-estimation of comorbidities estimated by the CCI scores based on the diagnosis in the claims database [3]. The ICD-10 code-based claims database diagnoses could be underestimated or overestimated in some cases. However, this would not significantly deteriorate the accuracy of AML diagnosis because research has confirmed that the accuracy of cancer diagnoses is higher than 92% using the NHIS claims database [43]. Another database-related limitation of our study is the accuracy of the mortality data in the claims database. Although claims data are known to have limitations in capturing mortality information because they only include hospital deaths, the death indicator of the NHIS has a true positive rate of 97% for high-risk cancers [44]. This study acknowledges a limitation regarding the assessment of the impact of the venetoclax plus HMA regimen on the elderly population. Although the combination of venetoclax and HMA represents a significant advancement in AML treatment since its introduction in 2019, this regimen has only been covered by insurance starting from February 2023 in South Korea. Consequently, the data reflecting the full potential benefits of this treatment are limited within the timeframe of our study. Future research, encompassing a longer observation period, is required to comprehensively evaluate the effects of venetoclax and HMA on the management and outcomes of AML in the elderly population.

## Conclusions

In summary, our study revealed an increasing age-specific incidence of de novo AML among the population aged 60 years or older and yet a decreasing trend in the annual ASIR derived from a nationally representative population database. The unadjusted survival rate worsened among patients older than 65 years; however, after adjustment in the Cox analysis, we observed an improvement in survival rates over the study period for the overall population. Our findings suggest that there is a pronounced need for further well-designed studies to validate the impact of expanded treatment options for older patients with AML and to explore strategies for improving treatment outcomes in this population.

## Supporting information

S1 Fig Study design and settings. (TIF)

S2 Fig Flow chart of study cohort selection for the estimation of incidence rate and survival. (TIF)

S1 File (DOCX)
