# Correction: Impact of aging on acute myeloid leukemia epidemiology and survival outcomes: A real-world, population-based longitudinal cohort study

**DOI:** 10.1371/journal.pone.0353299

**Published:** 2026-07-06

**Authors:** Hyun Jin Han, Kyungseon Choi, Hae Sun Suh

The second author’s name is spelled incorrectly. The correct name is: Kyungseon Choi.
