# Supplementary figures and images for: Impact of aging on acute myeloid leukemia epidemiology and survival outcomes: A real-world, population-based longitudinal cohort study
Source: PLoS One. 2024 May 21;19(5):e0300637. doi: 10.1371/journal.pone.0300637 (PMC11108202; doi:10.1371/journal.pone.0300637)

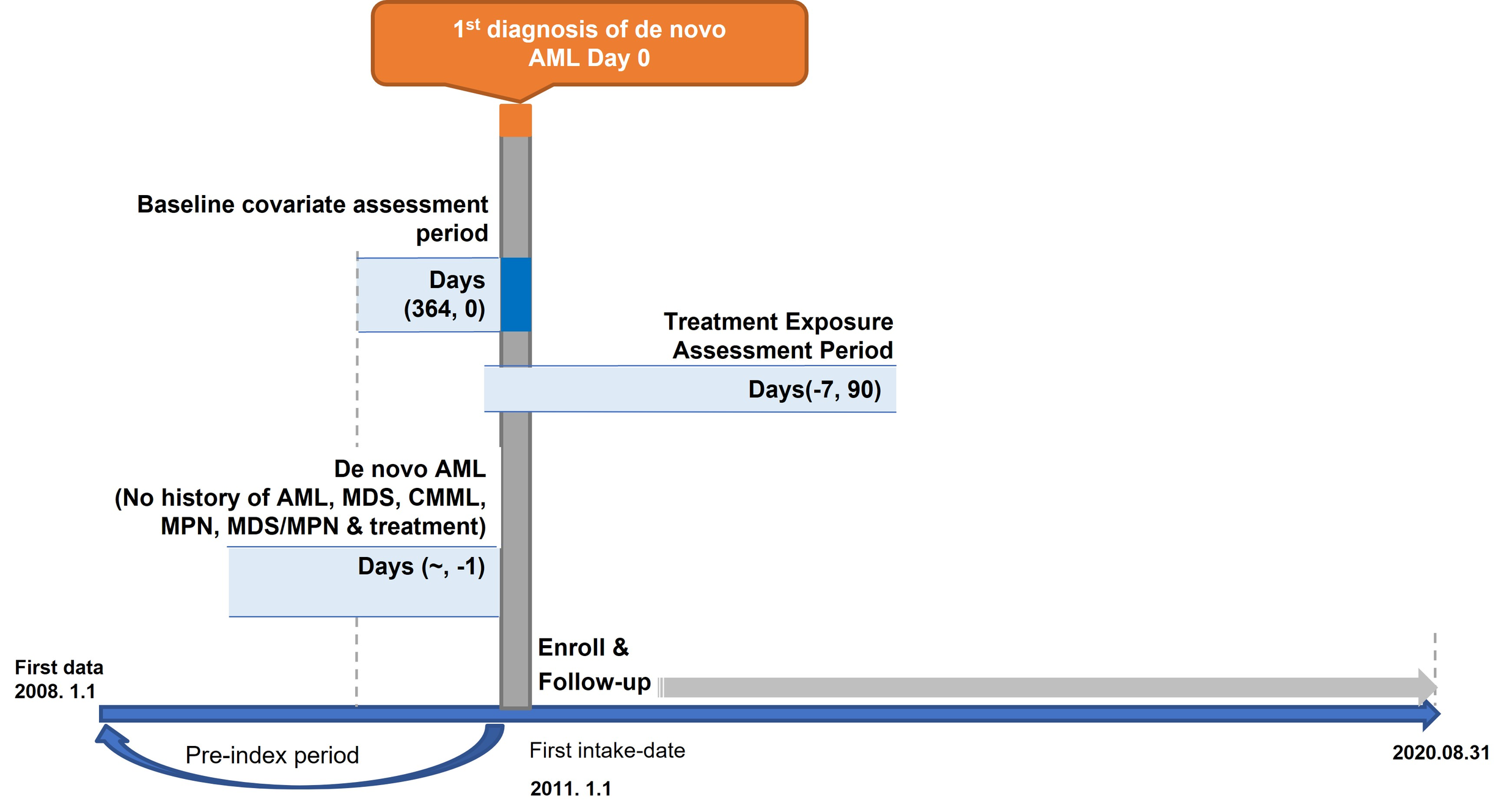

Supplement: S1 Fig — (TIF) [file pone.0300637.s001.tif]

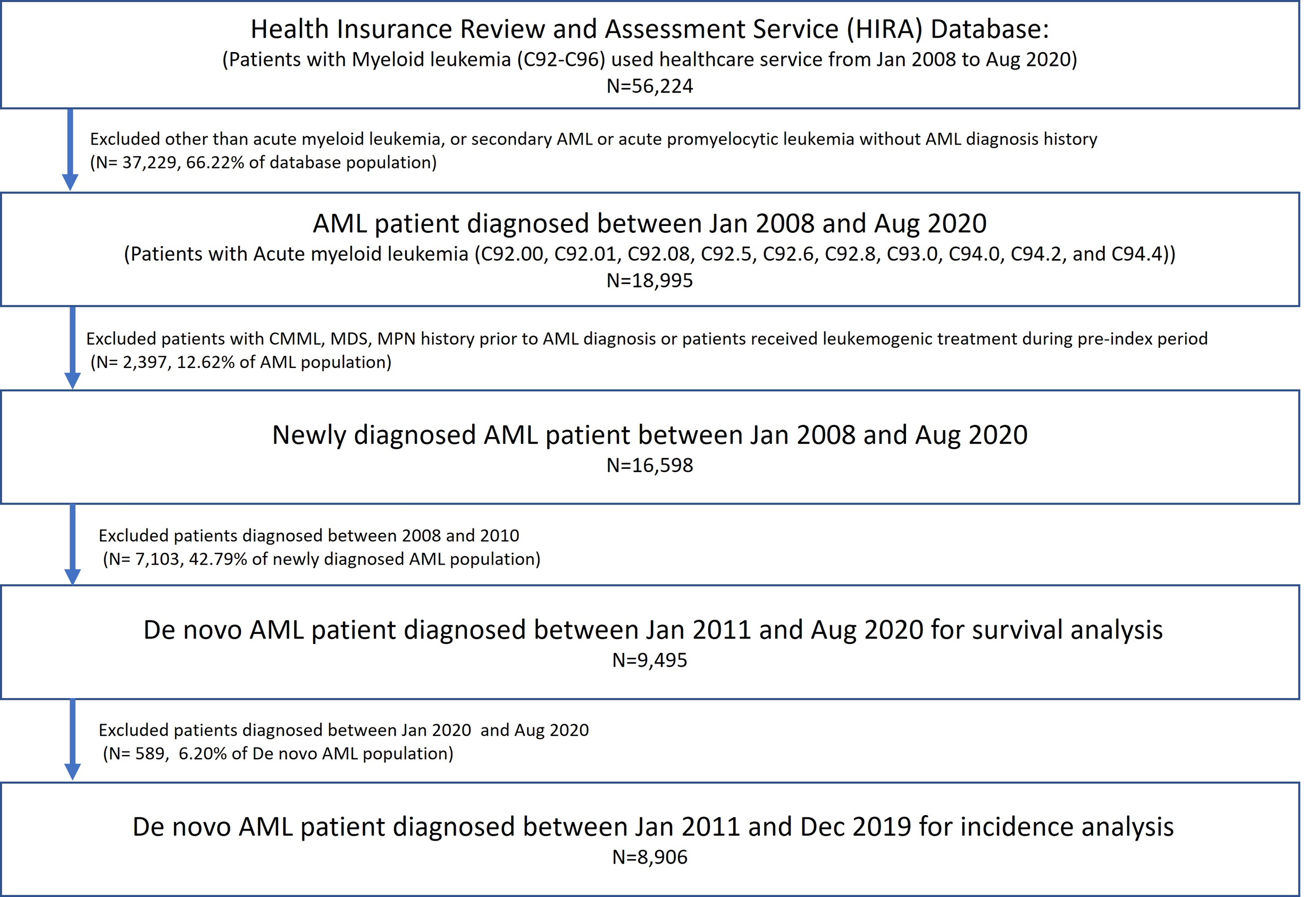

Supplement: S2 Fig — (TIF) [file pone.0300637.s002.tif]
