# Supplementary material for: Impact of aging on acute myeloid leukemia epidemiology and survival outcomes: A real-world, population-based longitudinal cohort study
Source: PLoS One. 2024 May 21;19(5):e0300637. doi: 10.1371/journal.pone.0300637 (PMC11108202; doi:10.1371/journal.pone.0300637)

**S1 Fig. Study design and settings**


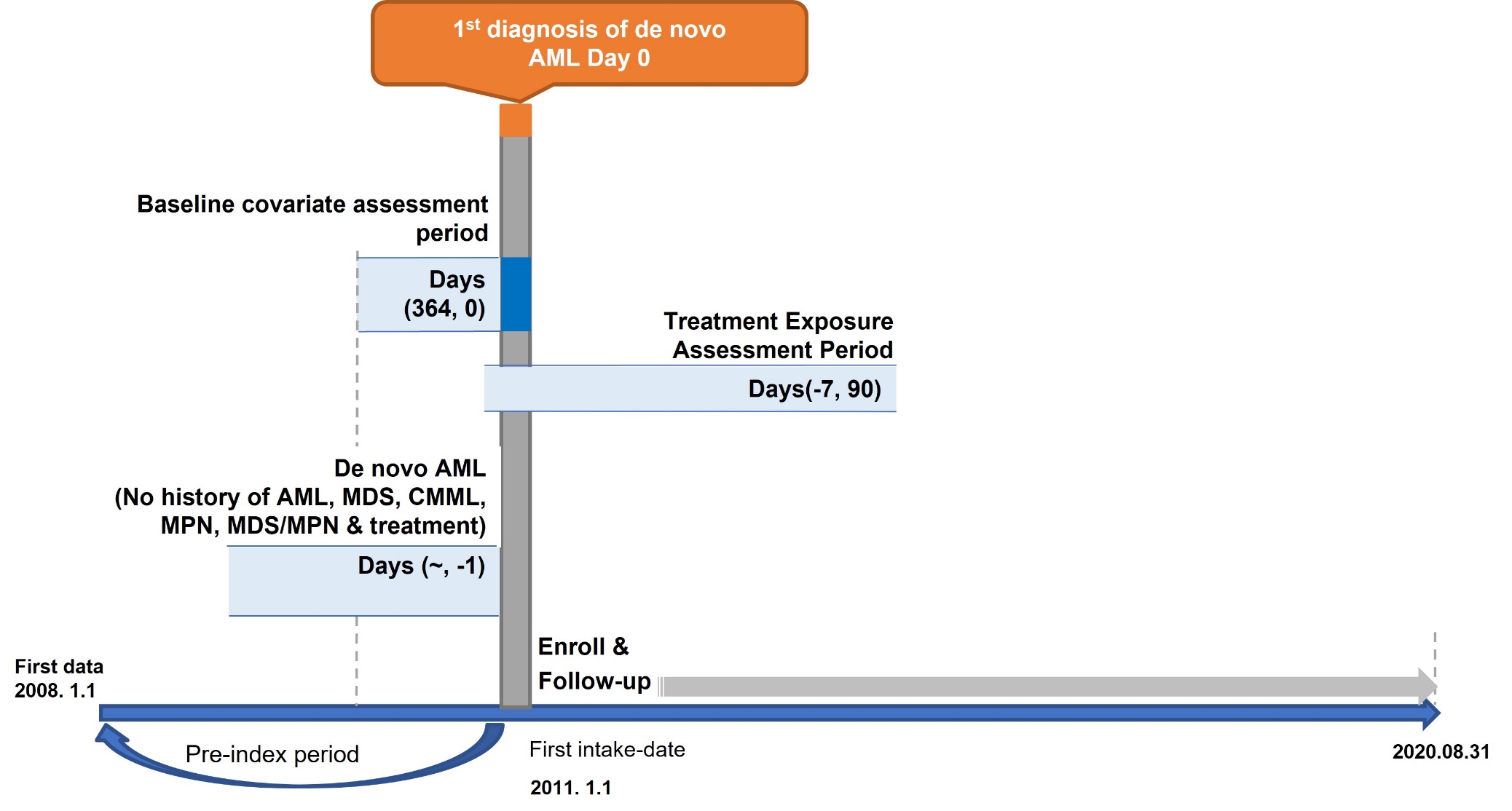


**S2 Fig. Flow chart of study cohort selection for the estimation of incidence rate and survival**


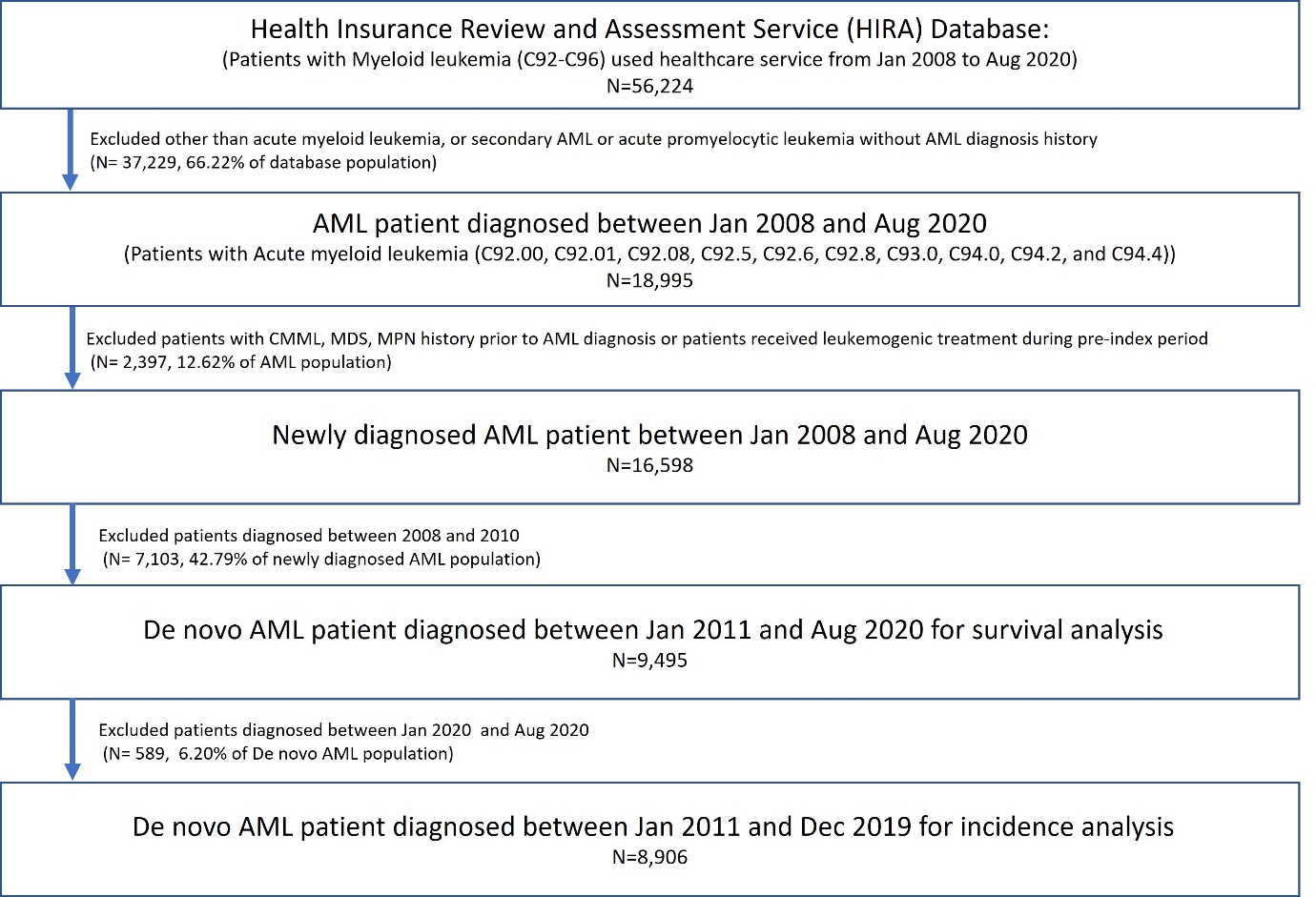

Supplement: S1 File — (DOCX) [file pone.0300637.s003.docx]
